# A Comparison of Wound Area Measurement Techniques: Visitrak Versus Photography

**Published:** 2011-04-18

**Authors:** Angela Christine Chang, Bronwyn Dearman, John Edward Greenwood

**Affiliations:** ^a^School of Medicine, University of Adelaide, Adelaide, South Australia; ^b^Adult Burn Centre, Royal Adelaide Hospital and Skin Engineering Laboratory, Hanson Institute, Adelaide, South Australia

## Abstract

**Objective:** To investigate whether a cheap, fast, easy, and widely available photographic method is an accurate alternative to Visitrak when measuring wound area in cases where a non–wound-contact method is desirable. **Methods:** The areas of 40 surgically created wounds on porcine models were measured using 2 techniques—Visitrak and photography combined with ImageJ. The wounds were photographed with a ruler included in the photographic frame to allow ImageJ calibration. The images were uploaded to a computer and opened with ImageJ. The wound outline was defined from the photographic image using a digital pad, and the ImageJ software calculated the wound area. The Visitrak method involved a 2-layered transparent Visitrak film placed on the wound and the outline traced onto the film. The top layer containing the tracing was retraced onto the Visitrak digital pad using the Visitrak pen and the software calculated the wound area. **Results:** The average wound area using the photographic method was 52.264 cm^2^ and using Visitrak was 51.703 cm^2^. The mean difference in wound area was 0.560 cm^2^. Using a 2-tailed paired *T* test, the *T* statistic was 1.285 and the value .206, indicating no statistical difference between the two methods. The interclass correlation coefficient was 0.971. **Conclusions:** The photographic method is an accurate alternative to Visitrak for measuring wound area, with no statistical difference in wound area measurement demonstrated during this study. The photographic method is a more appropriate technique for clean and uncontaminated wounds, as contact with the wound bed is avoided.

The measurement of wound dimension is an important component of successful wound management. Monitoring changes in wound area allows assessment of treatment efficacy and early detection of stasis or deterioration. The information can also aid in research, communication with patients and between practitioners, evidence-based clinical decision making, and, ultimately, an improvement in the quality of patient care. Wound surface area calculations have been shown to be a valid and accurate indicator of wound progress.^1^ Although measuring wound volume may provide additional information, it has received limited attention in the literature,^2^ possibly due to practical difficulties incumbent with volume measurement. Several series have demonstrated that wound circumference and area directly correlate to wound volume,^3^,^4^ and thus volume is not required for appropriate wound monitoring.

There are a wide variety of devices and methods available to measure wounds. However, to be useful in routine clinical practice, the technique needs to be time- and cost-efficient, accurate, sensitive, unbiased, comfortable for the patient, and useable by any operator.

The simplest method of determining wound area is by measuring with a ruler. The greatest length is multiplied by the perpendicular greatest width. This technique is quick, inexpensive, and easy to perform but assumes that the wound is square or rectangular in shape. This type of calculation has been shown to overestimate wound area by 10% to 44%,^5^,^6^ with accuracy decreasing as wound size increases.^6^,^7^ This method is imprecise and inappropriate for wounds which are large, irregular, or cavitous.^6^ Accuracy can be improved by using the formula for the area of an ellipse rather than a rectangle,^8^ or by using a formula for a shape similar to that of the wound.^9^

Manual tracing, by placing a transparent film over the wound and tracing the outline with a permanent marker allows more accurate area calculation when the wound is irregular. From the tracing, there are several methods of determining area. The tracing can be placed on a metric grid, and the number of squares of a known area counted. This is quick and easy,^10^ but inaccuracy arises when deciding the value of partial squares.^11^ The outline can also be cut out and weighed, although this may be time-consuming and error may arise during cutting and weighing. This also requires accurate scales capable of detecting acetate fragments of potentially very small mass. Digital planimetry involves the same process of wound tracing, but the outline is then retraced onto a digital tablet, which calculates the area. This has the advantage of quick, precise, and objective calculation, as well as revealing wound circumference. However, added scope for error occurs with retracing.^12^ The accuracy of area measurement from wound tracings depends on correct and consistent identification of the wound margins. Several studies have shown that the largest source of error is when determining wound borders, rather than the process of calculating the area traced.^4^,^13^ The reliability of this technique decreases with decreasing wound size.^7^ The Visitrak system (Smith & Nephew Wound Management, Inc, Largo, Florida) is an example of a digital planimetry system developed in response to the need of an easy, fast, and reliable tool for clinical use. The wound is traced onto a Visitrak grid sheet, which is then retraced onto the Visitrak digital pad, which automatically completes the area calculations. Visitrak has been shown to have excellent intra- and interoperator reliability, high validity, and a correlation co-efficient of 0.99 compared to other systems of digital planimetry.^14^ Its main advantages are the rapidity of the technique (one half to one third the time of other systems), and its relative inexpense compared to other planimetry systems. Difficulties using the Visitrak system are the same as those when manually tracing any wound onto transparent film—difficulty identifying wound margins, fogging under the film impeding vision, pressure on the wound altering the outline, stiff film unable to conform closely to wound surface, plus the requirement of film to be in contact with the wound risking contamination, wound bed damage, and patient discomfort and pain.^3^,^15^ The Visitrak and many other systems requiring on-wound tracing minimize the risk of contamination by providing a clean (although nonsterile) disposable sheet under the tracing sheet in contact with the wound.^16^ Although for nonsterile venous ulcers the risk of infection from a tracing grid is minimal, contamination becomes a major concern for patients presenting with burns and surgically “clean” wounds.

Photography can be used to calculate wound area. The borders of the wound are traced from a digital photograph using a mouse or digital pen on a digital tablet. A ruler, or other accurate scale, photographed near the wound allows the user to calibrate the software to enable it to judge distance. An advantage of photography is that it does not require contact with the wound. It provides a permanent record of not only wound size but also appearance. As with other forms of wound tracing, determining wound borders is subjective and may be problematic if edges are poorly defined or obscured by debris. High-resolution photographs are required to properly identify epithelial growth at wound margins.^17^ Important considerations when using photography are photo quality, lighting, and camera angle. Variation in camera angle can lead to an underestimation of wound area by up to 10%.^18^ Given that a 2D image is representing a 3D structure, apparent wound size discrepancy may also occur when tracing circumferential wounds, or those on curved body surfaces.^19^,^20^ Despite these difficulties, studies have still reported very good intra- and interoperator reliability.^6^,^11^,^21^ A variation of this system is photogrammetry, such as utilized in the VeV system (Vista Medical, Winnipeg, Mannitoba, Canada). This computerized system consists of a video camera, frame grabber, and customized wound assessment software. A digital photograph of the wound, including a target plate, is uploaded to the computer. The software uses the target plate for calibration and calculates the wound area. Computerized photogrammetry has proved to be more accurate than the manual method on both plaster of Paris models^22^ and animal wounds,^11^ although its clinical application may be limited by greater cost.

Sterophotogrammetry involves the use of 2 cameras and the computer to reconstruct 3D images of wounds allowing calculation of wound area and volume.^19^ Although sterophotogrammetry seems to be the most accurate method^22^,^23^ of determining wound area, its use requires additional training, the equipment is expensive, and it is time-consuming, making it impractical for routine clinical assessment.

The aim of this study was to investigate whether a cheap, fast, easy, and widely available photographic method is an accurate alternative to Visitrak, when measuring wound area in cases where a non–wound-contact method is desirable.

## METHODS

### Animal Subjects

Porcine models with surgically created wounds already in use for alternative research were used for this study. A total of 40 wounds (4 wounds on 2 swine measured once a week for 5 weeks) were measured using 2 methods—Visitrak and photography combined with ImageJ software. Each wound was created as an 8 × 8 cm^2^ dissected to the paniculus adiposus. All were located on the dorsum of the animal to minimize surface curvature and allow ease of photography. During measurement, the animals were anaesthetized to minimize movement. On each occasion, they were placed in the same position under consistent lighting conditions. Prior to measurement and photography, the wounds were cleaned and slough removed to provide easily visible wound margins. For either technique, wound border tracing was done by the same operator in every instance.

### Photographic method

The wounds were photographed using a Canon EOS 550D digital camera, with electro-focus short back focus (EF-S) 18-55 mm lens. A “square” ruler with markings on each side was placed around the wound and included in the photographic frame to allow calculation of wound dimensions once uploaded to the computer. Using a visual estimate, an effort was made to hold the camera at the zenith above the wound, and the lens parallel to the plane of the surface. The images were uploaded to a computer and opened with ImageJ, a Java imaging processing program available for free download from the Internet. For each photographic image, measurements were calibrated using the ruler at the base of the image included in the photographic frame. The wound outline was defined from the photographic image using a digital pad. Following tracing, the ImageJ software calculated the wound area.

### Visitrak method

**Figure 1:**
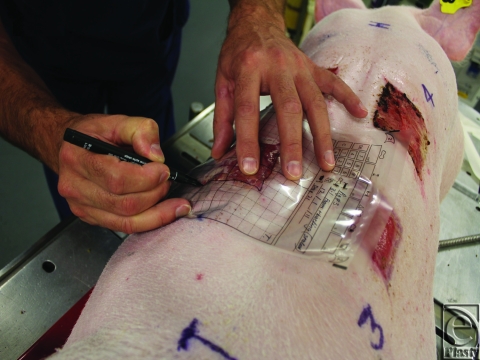
The method of tracing a wound outline onto a Visitrak transparent film.

The wounds were also measured using Visitrak, a digital planimetry system. A 2-layered transparent Visitrak film was placed on the wound (Fig 1), and the outline traced onto the film using a permanent marker. The layer of the film in contact with the wound was discarded. The top layer containing the tracing was retraced onto the Visitrak digital pad using the Visitrak pen and the software calculated the wound area.

### Data analysis

The data was compiled and analyzed using Microsoft Excel 2007 (Microsoft, Redmond, California).

## RESULTS

**Figure 2:**
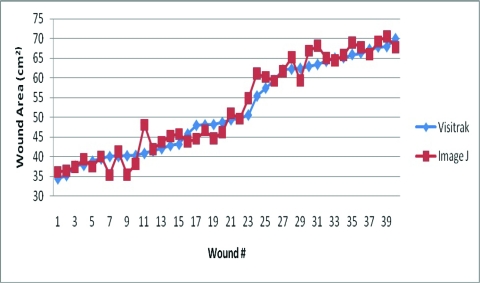
A comparison of wound area measurement using the Visitrak and photographic technique.

Figure 2 demonstrates the individual wound area surface measurements for all 40 wounds. The mean and variance of the surface area for both techniques are shown in Table 1. The average wound area using the photographic method was 52.264 cm^2^ and using Visitrak was 51.703 cm^2^. The mean difference in wound area between the two methods was 0.560 cm^2^. Analysis of the data, using a 2-tailed paired *T* test, revealed a *T* statistic of 1.285, with a *P* value of .206. Thus there was no statistical difference between the two methods when measuring wound area. The interclass correlation coefficient (ICC) was 0.971. Variance of the Visitrak method (129.851) was slightly lower than that of the photographic method (147.305).

## DISCUSSION

The results of previous wound area measurement studies comparing photographic and transparency tracing methods have fairly consistently shown that both yield statistically similar wound area results.^13^,^17^,^21^ The majority of these studies that were performed on patients rather than synthetic wound models have been measuring the area of venous ulcers, which have a limited size and body region distribution. Our results concur with previous series, showing the photographic method to be an accurate alternative to transparency tracings, in our case Visitrak. The high ICC of 0.971 in our study compares favorably to other studies of similar design where an ICC of 0.99^13^,^21^ was recorded.

### Wound tracing

**Figure 3:**
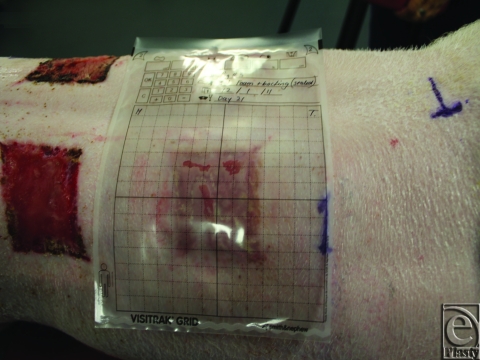
“Fogging” of the Visitrak film while tracing the wound outline.

**Figure 4:**
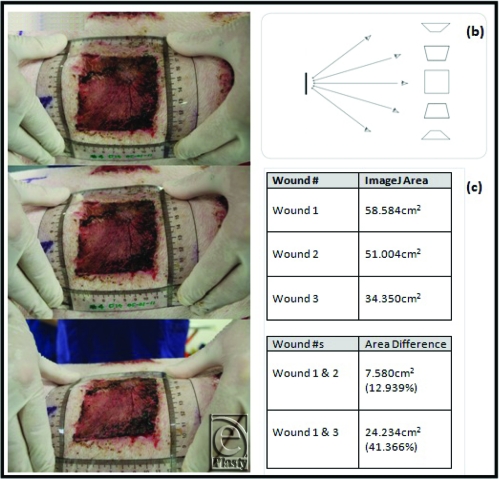
(*a*) The same wound photographed from varied camera angles demonstrating the effect of angle on observed size and shape. (*b*) Diagrammatic representation of a wound seen from varied angles. (*c*) ImageJ wound area calculations from the photographs in (*a*).

The usefulness of both methods depends on the accuracy of wound margin identification. Prior to tracing and photography, the wound margins were made as clear as possible. The wounds were undressed and any debris, slough, and necrotic tissue removed. The wound and surrounding tissue were then cleaned with saline and dried. A problem encountered while using the Visitrak system was fogging of the Visitrak tracing grid when placed on the wound (Fig 3). This only occurred when tracing moist wounds making identification of wound margins difficult. We attempted to hold the camera directly above the wound as camera angle affects the apparent measurable area (Fig 4). As mentioned previously, varied camera angle can lead to underestimation of wound area by up to 10%.^18^ To demonstrate this phenomenon, one of our wounds was photographed with varying camera angle and the area calculated using ImageJ (Fig 4). As expected, the wound area decreased as the angle from the zenith increased.

### Study limitations

The results should be interpreted within the limitations of the methodology. During this study, the two measuring methods were compared using wounds of a relatively constant size and shape, on a body region easy to photograph. Previous studies have demonstrated that the reliability Visitrak decreases with decreasing wound size.^8^ Likewise, the accuracy of photography is compromised by curved surfaces, tapering of limbs, and circumferential wounds.^24^

Intra- and interoperator consistencies were not tested. For both methods, each wound was measured only once; however, the tracings for each method were performed by the same operator. Previous studies have demonstrated high inter- and intraoperator reliability for Visitrak,^15^ but some have found considerable interoperator variability when tracing the wound outline from a photographic image.^17^

## CONCLUSION

The photographic method is an accurate alternative to Visitrak for measuring wound area, with no statistical difference in wound area measurement demonstrated during this study. The photographic method is a more appropriate technique for clean and uncontaminated wounds as contact with the wound bed is avoided, negating the risk of wound contamination, wound bed damage, and patient discomfort.

## ACKNOWLEDGMENT

The authors would like to thank Dr David Butler, University of Adelaide, for his statistical advice and assistance.

## Figures and Tables

**Table 1.** Statistical Results Comparing the Visitrak and Photographic Method

	Photo + ImageJ	Visitrak
Mean	52.263925	51.7025
Variance	147.3046944	129.8505064
Observations	40	40
Pearson correlation	0.974376635	
Hypothesized mean difference	0	
Degrees of freedom	39	
*t* Statistic	1.284814729	
*P* (*T* ≤ *t*) 1–tail	.103217924	
*t* Critical 1–tail	1.684875122	
*P* (*T* ≤ *t*) 2–tail	.206435848	
*t* Critical 2–tail	2.022690901	
